# A molecular, morphological, and physiological comparison of English and German populations of *Calliphora vicina* (Diptera: Calliphoridae)

**DOI:** 10.1371/journal.pone.0207188

**Published:** 2018-12-03

**Authors:** Kwankamol Limsopatham, Martin J. R. Hall, Richard Zehner, Barbara K. Zajac, Marcel A. Verhoff, Narin Sontigun, Kom Sukontason, Kabkaew L. Sukontason, Jens Amendt

**Affiliations:** 1 Institute of Legal Medicine, Goethe University, Frankfurt, Germany; 2 Department of Parasitology, Faculty of Medicine, Chiang Mai University, Chiang Mai, Thailand; 3 Department of Life Sciences, Natural History Museum, London, England; North Carolina State University, UNITED STATES

## Abstract

The bluebottle blow fly *Calliphora vicina* is a common species distributed throughout Europe that can play an important role as forensic evidence in crime investigations. Developmental rates of *C*. *vicina* from distinct populations from Germany and England were compared under different temperature regimes to explore the use of growth data from different geographical regions for local case work. Wing morphometrics and molecular analysis between these populations were also studied as indicators for biological differences. One colony each of German and English *C*. *vicina* were cultured at the Institute of Legal Medicine in Frankfurt, Germany. Three different temperature regimes were applied, two constant (16°C & 25°C) and one variable (17–26°C, room temperature = RT). At seven time points (600, 850, 1200, 1450, 1800, 2050, and 2400 accumulated degree hours), larval lengths were measured; additionally, the durations of the post feeding stage and intrapuparial metamorphosis were recorded. For the morphometric and molecular study, 184 females and 133 males from each *C*. *vicina* population (Germany n = 3, England n = 4) were sampled. Right wings were measured based on 19 landmarks and analyzed using canonical variates analysis and discriminant function analysis. DNA was isolated from three legs per specimen (n = 61) using 5% chelex. A 784 bp long fragment of the mitochondrial cytochrome b gene was sequenced; sequences were aligned and phylogenetically analyzed. Similar larval growth rates of *C*. *vicina* were found from different geographic populations at different temperatures during the major part of development. Nevertheless, because minor differences were found a wider range of temperatures and sampling more time points should be analyzed to obtain more information relevant for forensic case work. Wing shape variation showed a difference between the German and English populations (*P*<0.0001). However, separation between the seven German and English populations at the smaller geographic scale remained ambiguous. Molecular phylogenetic analysis by maximum likelihood method could not unambiguously separate the different geographic populations at a national (Germany vs England) or local level.

## Introduction

Forensic entomology, the interpretation of insect evidence in legal investigations, became popular in many countries as an important forensic tool at the beginning of the twenty-first century [1–3]. Sampling and identifying insects from a body, mainly species from the orders Diptera (flies) and Coleoptera (beetles), is helpful to estimate the minimum time since death (or minimum post-mortem interval) by evaluating the insect succession or by calculating the age of developing insects on a body [4]. Size (e.g. length or weight) and specific developmental events (e.g. 1^st^ larval moult/ecdysis or, later on, the pupariation) are two measures which, eventually, lead to an age determination by isomegalen [5], isomorphen [6, 7], curvilinear regression [8] or accumulated degree hours or days (ADH/ADD) methods [8]. Blow flies (Diptera: Calliphoridae) are typically both the first colonizers of a cadaver and the most common insects associated with dead animals and humans and, hence, are the focus of many studies in forensic entomology [9]. Published development data act as references for the growth rate and enable an age estimation of the immature stages. It is important to determine whether populations of the same species show different rates of development related to their geographical origin [10]. Such population or geographic specificity would hamper the use of reference data in forensic entomology when originating from locations different to the crime scene.

Morphometrics are defined as the quantitative studies of biological size and shape, shape variation, and its covariation with other biotic or abiotic factors [11] and can be valuable tools for inter- and even intraspecific discrimination. In recent years, a landmark-based geometric morphometric analysis of insect wings has been extensively applied in entomology, particularly in taxonomy [12–14] and for comparing geographical populations of species [15, 16]. Examples are the discrimination of the blow flies *Cochliomyia hominivorax* and *C*. *macellaria* [17] or *Chrysomya albiceps* and *C*. *megacephala* [18]. Wing morphometrics has also been applied to differentiate 11 species of *Anopheles* (*Nyssorhynchus*) [19]. Hall et al. [16] have reported that using wing morphometry showed significant difference between populations of the Old World screwworm fly, *C*. *bezziana*, from Africa (Tanzania, South Africa Sudan, Zaire, Zimbawe) and Asia (Sumba, Indonesia).

Beside morphometric tools, species identification based on molecular analysis is common practice in forensic entomological case work [20]. Such DNA-based methods usually focus on the mitochondrial (mtDNA) rather than the nuclear genome because of its high copy number, lack of introns, its limited exposure to recombination and its haploid mode of inheritance [21], therefore, having an increased chance of generating species-specific markers [22]. The mitochondrial cytochrome c oxidase subunit I (COI) gene has been established as the DNA marker of choice in forensic entomology to identify many relevant Diptera species [23, 24]. Another mitochondrial region of interest, especially when establishing phylogenetic links between various species, is a fragment of the gene coding cytochrome b (cyt b) [25].

*Calliphora vicina*, commonly called the bluebottle fly, is a very common blow fly species distributed throughout the Palaearctic region that plays an important role as forensic evidence in crime investigations [3, 26]. Several studies analyzed its growth for populations from Austria, Canada, Germany, Russia, the UK, and the USA [4, 9, 27]. The locations from where the analysed flies were derived represent a broad range of habitats in the northern hemisphere. However, Hwang and Turner [28] showed the existence of even a small scale variation and phenotypic plasticity for *C*. *vicina* as the consequence of the warming of an urban area in London (UK), therefore some might argue that using reference data derived from, e.g., Russia cannot be used to estimate the age of, e.g., an Austrian population, because of adaptations due to local climate conditions. The main aim of this study was, therefore, an intermediate approach to compare two relatively close geographically but clearly separated populations. We analysed for the first time developmental rates of two geographically distinct *C*. *vicina* populations from Germany and England under the same temperature regimes in the same laboratory. In addition, we evaluated the wing venation and cytochrome b sequence of flies from different locations of England and Germany to check the potential for these tools to discriminate specimens from both regions, thereby introducing a marker(s) that could indicate possible biological differences.

## Materials and methods

### Fly specimens

**Fig 1:**
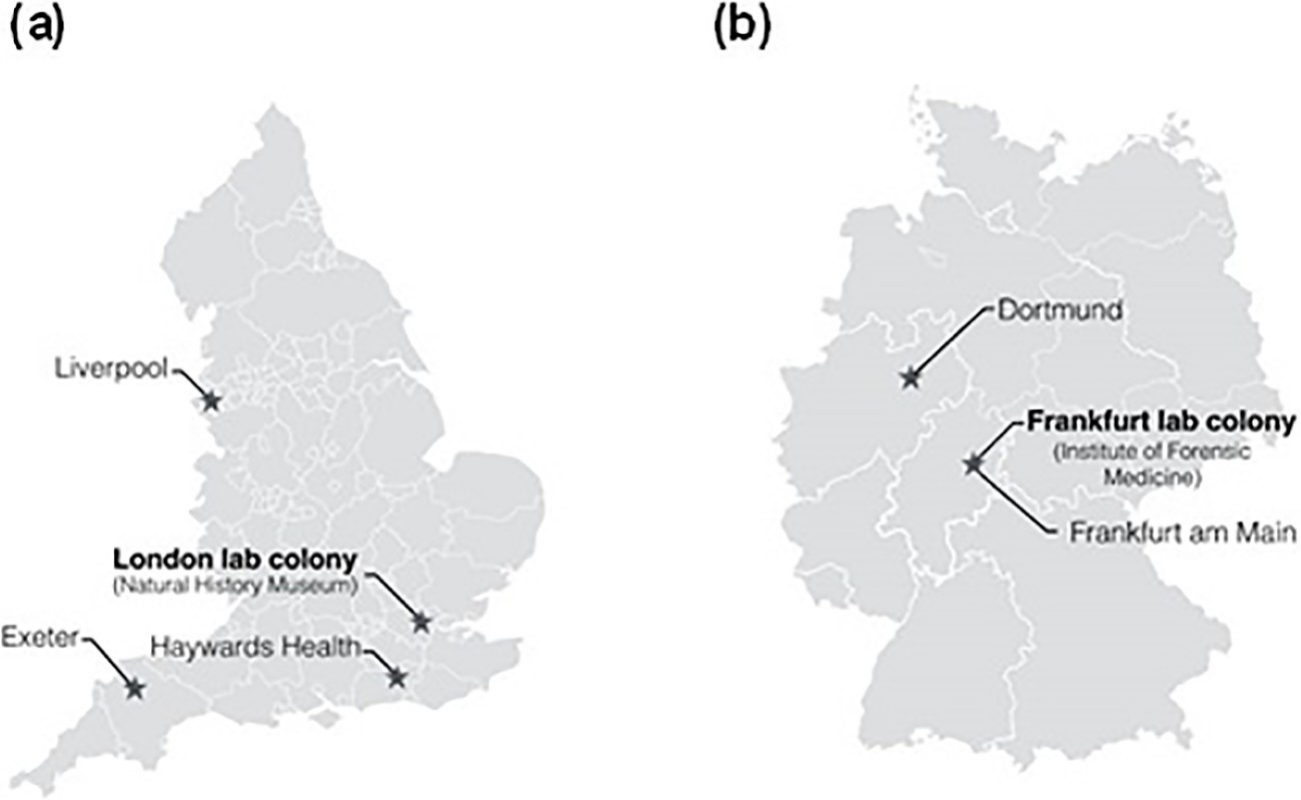
Map of England (a) and Germany (b) showing the sampling areas.

For wing morphometrics and molecular analysis, adult flies of *C*. *vicina* were first classified into two populations; German flies and English flies. The German population was further divided into 3 groups: Frankfurt am Main (coded as CVG1), Dortmund (CVG2) and Frankfurt laboratory colony (CVG3); the latter had been maintained for about 1.5 years without refreshment, i.e., no new specimens from the field. The English population was divided into 4 groups: Exeter (coded as CVE1), Haywards Heath (CVE2), Liverpool (CVE3) and London laboratory colony (CVE4), which had been in colony for less than six months. The geographic location of these samples is shown in Fig 1 and Table 1.

**Table 1:** Geographic locations of the sampled areas and number of specimens for wing morphometric and for cytochrome b gene analyses.

				For wing morphometric analysis			For cytochrome b gene analysis		
Populations	Code	Latitude (S)	Longitude (W)	Female	Male	Total	Female	Male	Total
**German**									
Frankfurt am Main	CVG1	50.1109° N	8.6821° E	32	17	49	4	9	13
Dortmund	CVG2	51.5136° N	7.4653° E	4	5	9	3	4	7
Laboratory colony, Frankfurt am Main	CVG3	50.1109° N	8.6821° E	50	50	100	4	5	9
**English**									
Exeter	CVE1	50.7184° N	3.5339° W	14	6	20	2	2	4
Haywards Heath	CVE2	50.9990° N	0.1063° W	18	2	20	1	12	13
Liverpool	CVE3	53.4105° N	2.9779° W	16	3	19	2	6	8
Laboratory colony, London	CVE4	51.5074° N	0.1278° W	50	50	100	3	4	7

### Developmental study

#### Fly rearing

Two established colonies of *C*. *vicina*, one from Frankfurt (Germany) and one from London (England), were processed as follows. Adult flies were held in rearing cages at room temperature (average temperature approximately 20°C, 79% RH) and a 12:12 L:D cycle. They were provided with water and sugar *ad libitum*. A piece of fresh pork liver was regularly placed into the cage as a protein source. At the beginning of each experimental run, the liver was checked for eggs 3 hours after placement. Resulting eggs were transferred into an incubator (LinTek MKKL 600/2), set at 25±1°C. Twenty-four hours after transferring the eggs into the incubator, hatched larvae were used for further experiments.

#### Experimental set-up and sampling

Specimens of each geographic population were exposed to 3 different temperature regimes, two constant (16°C & 25°C) and one fluctuating (17–26°C, Room temperature = RT). For each temperature, five groups of 20 freshly hatched larvae were transferred from the oviposition medium to approximately 20 g mixed minced meat (pork/beef) in a plastic cup, with each group in a separate cup. These cups were placed in 12 cm × 12 cm × 8 cm plastic containers which were filled with 2 cm of sawdust, serving later in development as the medium for pupariation. This protocol was repeated three times, leading to the analysis of 300 larvae (3 x 100) per temperature and geographic region.

The plastic containers with cups were moved daily within the incubators to avoid possible incubator-specific effects. At seven time points (ADH: 600, 850, 1200, 1450, 1800, 2050, 2400, based on a lower developmental threshold of 0 °C), 10 larvae (2 per container) were randomly selected, killed with hot water, and their length measured by using a geometrical micrometer [29]. Additionally, the start of the post-feeding stage, pupariation (white puparia visible) and emergence of adult flies were checked daily.

### Wing morphometrics

#### Wing preparation

**Fig 2:**
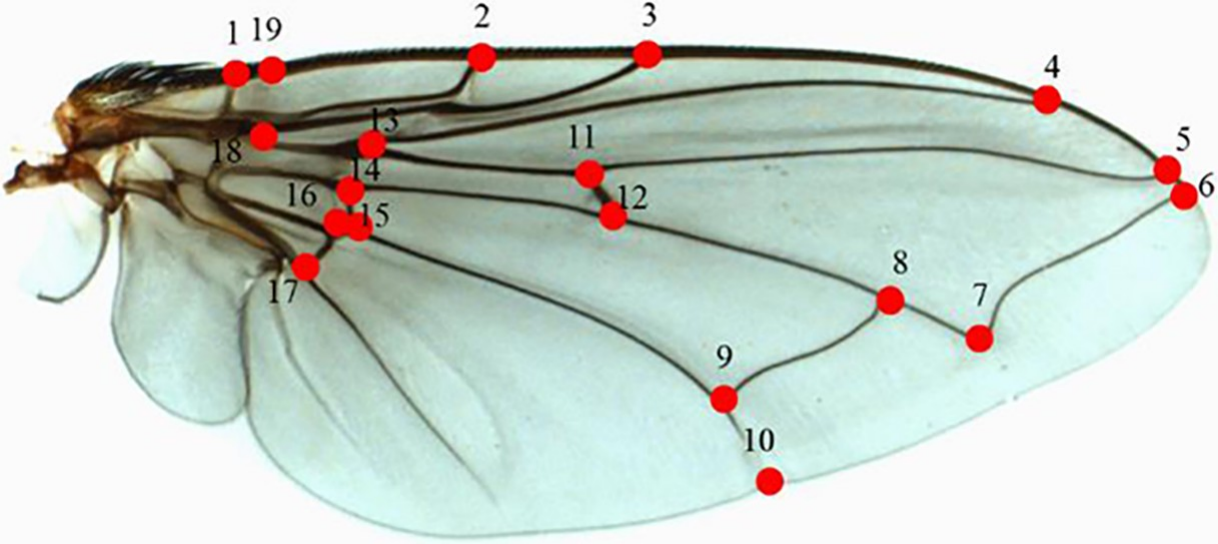
Right wing of *C*. *vicina* showing the 19 landmarks based on Hall et al. [16].

Fine forceps were used to remove the right wing of each fly. Each wing was placed on a drop of Euparal (Waldeck GmbH & Co. KG, Muenster Germany) on a glass slide, then a thin layer of Euparal was added to the wing and a cover slip was placed on top. After drying at room temperature for 24 h, each wing was photographed using a digital camera (AxioCam ICc1) attached to a stereomicroscope (Olympus SZ61) at 1×magnification. Tps files of each wing were created using TpsUtil V.1.70 software [30] to minimize bias in digitizing landmark locations. The coordinates of 19 landmarks were digitized using TpsDig2 V.2.26 software [31]. They were located at the base of the wing, the intersection of wing veins with each other, the intersections of wing veins with the wing margin, and the intersection of the cross vein with the major vein branch point (Fig 2). Digitization was undertaken twice in order to reduce the measurement error [32].

#### Wing morphometrics

The established tps files contained digitized coordinates of 19 homologous landmarks from all wings and were subjected to the MorphoJ software [33] for alignment using Procrustes Fit function. This alignment removed scale, position and rotation. The distances between each of the 19 landmarks were measured. For determining measurement error, the resulting coordinates of each specimen were averaged after a generalized Procrustes analysis in MorphoJ. Centroid size (square root of the sum of the squared distances between the center of the configuration of landmarks and each individual landmark) [34] was also averaged for each specimen.

For wing size variation analysis, the overall measure of wing size was estimated by the centroid size. As a normality test of centroid size showed non-normal distribution, wing size difference among species was evaluated by Kruskal-Wallis test (significance level 0.05). Furthermore, MorphoJ software was used to determine wing shape variation. The variation in the shape space was assessed using a principal component analysis (PCA) to display the major features in a dataset and discover patterns in the relations among specimens. Canonical variates analysis (CVA) was used to determine the most important shape characteristics for discriminating between groups (Germany and England) and among multiple groups of specimens (7 groups; Germany: Frankfurt am Main, Dortmund & Frankfurt laboratory colony; England: Exeter, Haywards Heath, Liverpool, and London laboratory colony). The statistical significance of pairwise differences in mean shapes was tested using permutation tests (10,000 replications) with Mahalanobis distances and Procrustes distances. Additionally, discriminant function analysis (DFA) and cross-validation test was used to confirm the individuals to the correct geographic regions. An UPGMA (unweighted pair-group method with arithmetic average) dendrogram was constructed based on Mahalanobis distances from CVA using PAST V.3.09 software (http://folk.uio.no/ohammer/past/) to observe phenetic relationships among 7 locations.

### Molecular analysis

#### Specimens

Adult *C*. *vicina* were collected using chicken liver baited fly traps (Red Top) and hand-held sweep nets in Germany and England. Geographic locations and number of specimens are shown in Table 1. Specimens were identified and then stored in 95% ethanol.

#### DNA extraction

Genomic DNA was isolated from three legs per specimen using 5% chelex suspension. DNA isolation was performed in a 1.5 ml reaction tube. 200 μl of 5% chelex-suspension was added, the sample homogenized, and mixed thoroughly by vortex. Homogenized samples were incubated at 50±5°C for 15 min, vortexed, heated at >100°C for 8 min in a heat block, and centrifuged at 13,000 rpm/15,000 ×g for 5 min. Isolated DNA was stored at -20°C until processing.

#### PCR

Only those specimens that generated a reasonable amount of DNA (n = 61) were further analysed. Here, a 784 bp long fragment of the mitochondrial cytochrome b gene was amplified using PCR Primers CB_PDR-WR04 (5’-ATTTCACgCTCATTAACT) and CB_CAL-F07 (5’-gTWATAggAACAgCTTTTATRgg) previously published by Ready et al. [35]. PCR reactions were performed in a Biometra T3000 thermocycler. Each 25 μl PCR reaction contained 10× PCR buffer, 0.2 mM dNTPs, 0.5 mM of each primer, 1 U of Taq DNA polymerase (Sigma–Aldrich), 1 μl DNA template, and nuclease-free water. PCR products were visualized by gel electrophoresis in a 2.5% agarose gel, stained with GelRed (Biotium, Darmstadt, Germany). The thermal cycler conditions were 3 min at 94°C, followed by five cycles of the following profile: 94°C for 30 s, 46°C for 30 s and 72°C for 1:30 min, followed by 30 cycles of 94°C for 30 s, 50°C for 30 s, and 72°C for 1: 30 min, with a final extension step of 72°C for 8 min.

#### Sequencing

Sequencing reactions were conducted using a BigDye Terminator v3.1 Cycle Sequencing Kit (Life Technologies) following a slightly modified manufacturer’s protocol. The sequencing reaction was performed using only 1 μl BigDye Ready Reaction Mix. Thermal cycling conditions were 28 cycles of the following profile: 96°C for 10 s, 50°C for 5 s, and 55°C for 4 min. Sequencing reactions were purified using gel-filtrated columns (Sigma–Aldrich, SigmaSpin Sequencing Reaction Clean-Up) following the manufacturers protocol. Purified Sequencing reactions were run on an ABI3130 genetic analyzer (Life Technologies).

### Statistics and phylogenetic analysis

#### Development

Statistical analysis was conducted in SPSS V.22.0 software (SPSS Inc., Chicago IL). Length differences of specimens for each temperature at seven time points was analysed by Kruskal-Wallis test, the times for reaching the various stages of development (post feeding, puparial, and adult stage) were compared by Chi-Square test. Differences were accepted as statistically significant if *P*<0.05.

#### Wing morphometrics

The tps files containing digitized coordinates of 19 homologous landmarks from all wings were subjected to the MorphoJ software [33] and then aligned using Procrustes Fit function to remove scale, position and rotation. For determining measurement error, the resulting coordinates of each specimen were averaged after a generalized Procrustes analysis in MorphoJ. Centroid size (square root of the sum of the squared distances between the center of the configuration of landmarks and each individual landmark) [34] was also averaged for each specimen.

For wing size variation analysis, the overall measure of wing size was estimated by the centroid size. The normality test of centroid size showed non-normal distribution, so wing size difference among species was evaluated by Kruskal-Wallis test. Furthermore, MorphoJ software was used to determine wing shape variation. The variation in the shape space was assessed using a principal component analysis (PCA). Canonical variates analysis (CVA) was used to determine the important features discriminating between groups. The statistical significance of pairwise differences in mean shapes was tested using permutation tests (10,000 replications) with Mahalanobis distances and Procrustes distances. Additionally, discriminant function analysis (DFA) and cross-validation test was used to confirm the individuals to the correct geographic regions. An UPGMA (unweighted pair-group method with arithmetic average) dendrogram was constructed based on Mahalanobis distances using PAST V.3.09 software (http://folk.uio.no/ohammer/past/).

#### Phylogenetic analysis

Quality control and assembly of sequences were performed using CodonCode Aligner (V. 5.1.1, CodonCode Corporation). Alignments and phylogenetic analysis were conducted with MEGA 6 (Center for Evolutionary Medicine and Informatics) [36]. Tree construction was performed using the Maximum Likelihood (ML) method with the Tamura 3-parameter model [37]. 1000 bootstrap replicates were used to test the reliability of the constructed tree. The tree was rooted with two Muscidae: *Stomoxys calcitrans* (DQ533708.1) and *Stomoxys uruma* (EU851371.1) as outgroups.

## Results

### Developmental study

**Fig 3:**
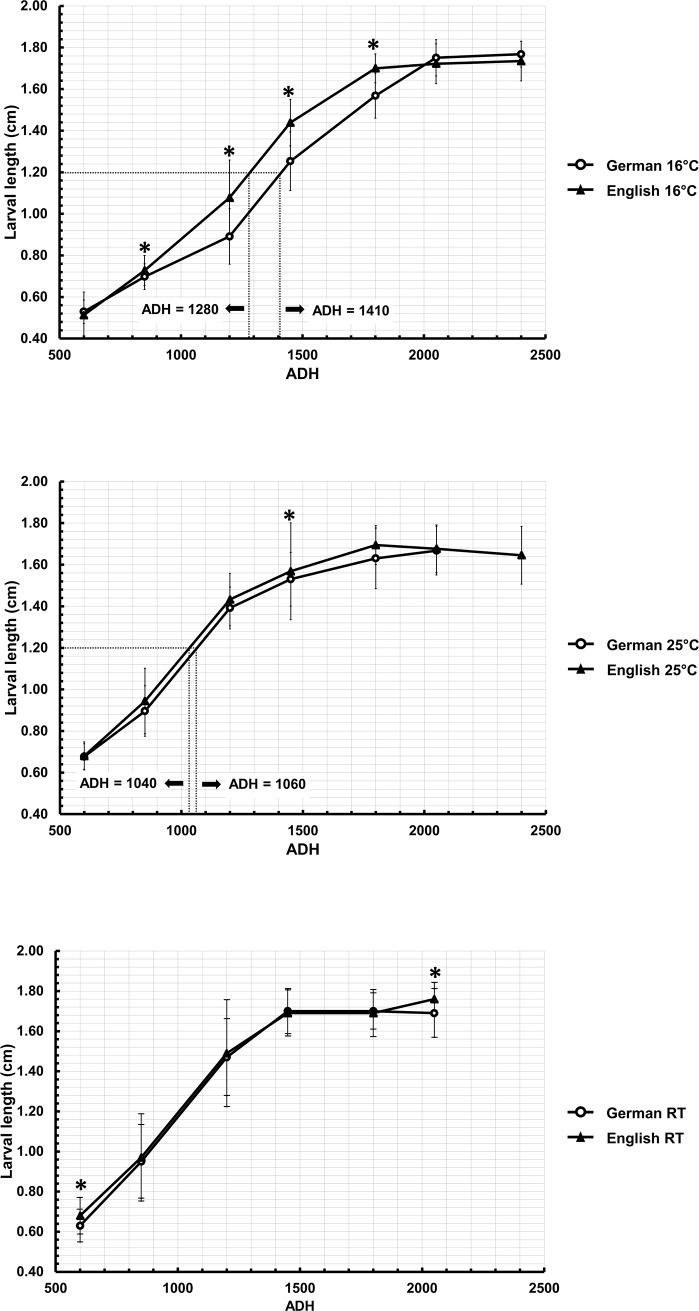
Comparison of developmental rate (larval length against ADH) between German and English *C*. *vicina* at three different temperatures; 16°C, 25°C, and RT = Room temperature; ADH = Accumulated Degree Hours, which was calculated using a basis of 0°C, for seven sampling points (ADH: 600, 850, 1200, 1450, 1800, 2050, and 2040). Dotted lines at larval length 1.2 cm (16°C and 25°C) indicate the exemplary age estimation via ADH, leading to different ages according to temperature conditions and/or population. For instance, German *C*. *vicina* took about 88 h (1410 ADH/16°C) to reach a length of 1.2 cm, whereas English *C*. *vicina* took about 80 h (1280 ADH/16°C); * = significant difference according to Kruskal Walis (*P<0*.*05*).

The development of *C*. *vicina* at a constant 16°C showed differences in average larval length between both populations at 850, 1200, 1450 and 1800 ADH, with English specimens significantly longer than their German conspecifics (Fig 3; S1 Data). However, at 25°C larval length of the English specimens was significantly longer only at 1450 ADH (Fig 3; S1 Data). Furthermore, populations reared at fluctuating temperatures showed significant differences in average larval length only at 600 and 2050 ADH (Fig 3; S1 Data).

**Fig 4:**
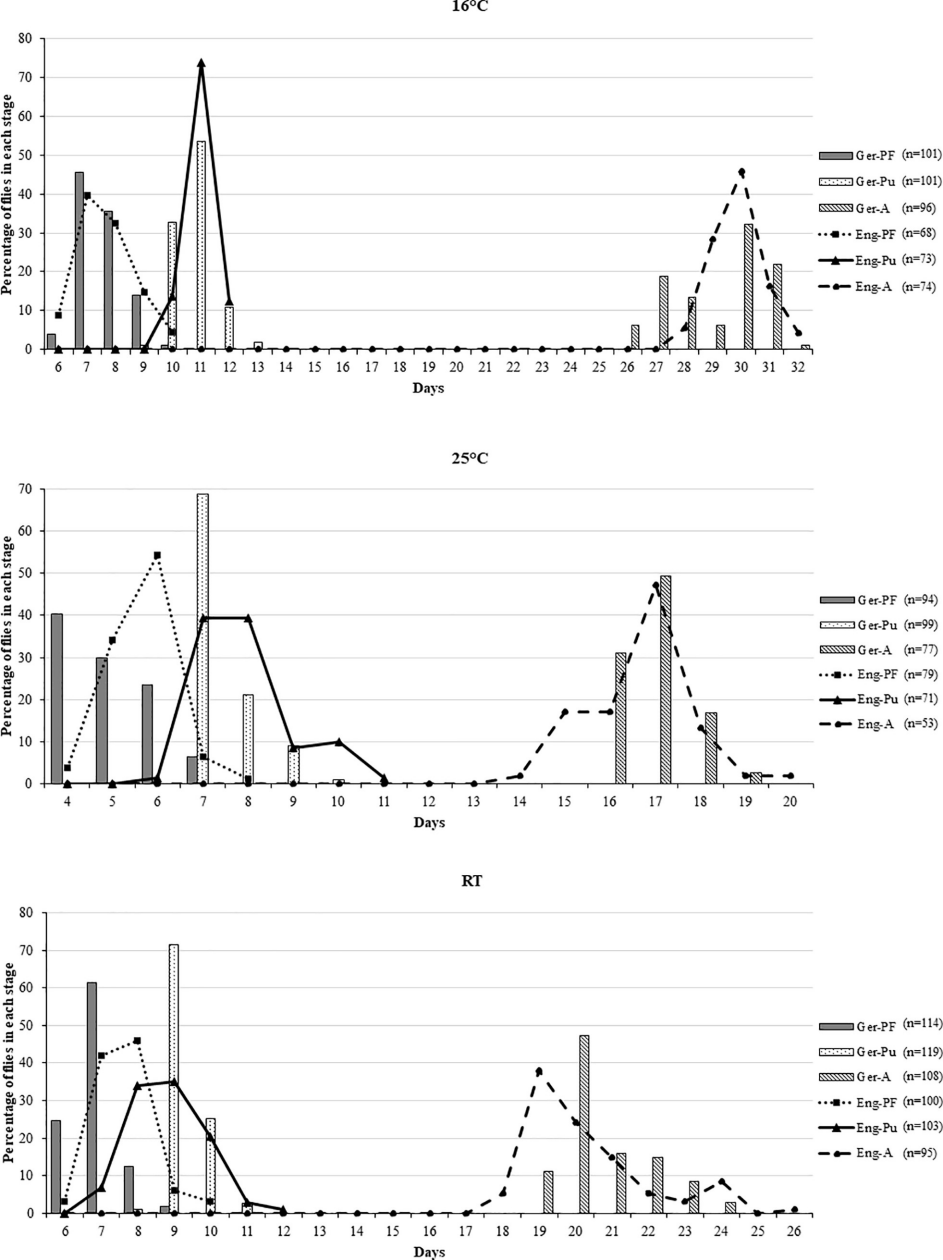
Percentage of *C*. *vicina* to reach the post-feeding (PF), puparial (Pu), and adult (A) stages at three different temperatures (25°C, 16°C, and RT).

*C*. *vicina* from both countries showed differences in time (days) to reach the post-feeding (Pf), puparial (Pu), and adult (A) stages at almost all experimental temperatures: only the durations to the post-feeding stage at 16°C were similar (6–10 days; Fig 4; S1 Data). Ger-Pu showed a similar peak of pupariation on days 10 and 11, but Eng-Pu reached their peak on day 11. At 25°C, Ger-Pf and Eng-Pf were reached on the same day (day 4) but German flies did this in a much higher percentage than the English flies, the English peak being on day 6. Although the English population began to pupariate earlier (day 6) at 25°C than the German samples (day 7), their highest peak occurred equally on days 7 and 8. A similar trend was seen under RT–the developmental times showed a slightly earlier pupariation of English (day 7) than German (day 9) populations and also earlier adult emergence of English (day 18) than German (day 19) flies. However, in relation to whether German or English flies reached the different development stages faster (= first), such mean differences were never greater than 0.8 days (Table 2; S1 Data).

**Table 2:** Mean time (days; n = 3 replicates) for *C*. *vicina* to reach the post-feeding, puparial, and adult stages.

Stages	German			English		
	25°C	16°C	RT	25°C	16°C	RT
Post-feeding	4.957	7.624	6.912	5.671	7.662	7.640
Pupa	7.424	10.802	9.294	7.901	10.986	8.816
Adult	16.909	29.094	20.743	16.660	29.851	20.263

### Wing morphometrics

#### Wing size variation

The different populations (CVG1-3 and CVE1-4) showed significant wing size variations. The mean ±SD of centroid size was 1227.04±103.88 (CVG1-3) and 1268.80±99.82 (CVE1-4). For the effect of gender on size, both populations showed a significant difference (*P*<0.05) between male and female. The mean of centroid size ±SD in German flies (CVG1-3) was 1157.04±80.62 (male) and 1285.65±82.94 (female), whereas for English flies (CVE1-4) it was 1217.95±77.21 (male) and 1300.45±99.52 (female). Furthermore, wing size measured by centroid size ±SD in each location showed significant differences between CVG1-CVG2, CVG2-CVG3, CVG1-CVE4 and CVG3-CVE4 (Tables 3 and 4; S2 Data)

**Table 3:** Centroid size±SD of *C*. *vicina* in 7 sampling areas.

Countries	Locations	Centroid size±SD
Germany	CVG1	1213.72±137.98
	CVG2	1304.76±69.18
	CVG3	1226.58±82.83
England	CVE1	1234.90±112.21
	CVE2	1249.59±100.73
	CVE3	1248.47±121.65
	CVE4	1283.28±90.84

**Table 4:** Statistical analysis comparison of wing size variation of *C*. *vicina* in 7 sampling areas.

Countries	Location	CVG2	CVG3	CVE1	CVE2	CVE3	CVE4
Germany	**CVG1**	0.047*	0.588	0.643	0.459	0.889	0.002*
	**CVG2**		0.010*	0.059	0.187	0.192	0.545
	**CVG3**			0.588	0.275	0.226	0.000*
England	**CVE1**				0.945	0.673	0.083
	**CVE2**					0.888	0.260
	**CVE3**						0.270

*significant difference, Kruskal-Wallis test (*P*<0.05)

#### Wing shape variation

**Fig 5:**
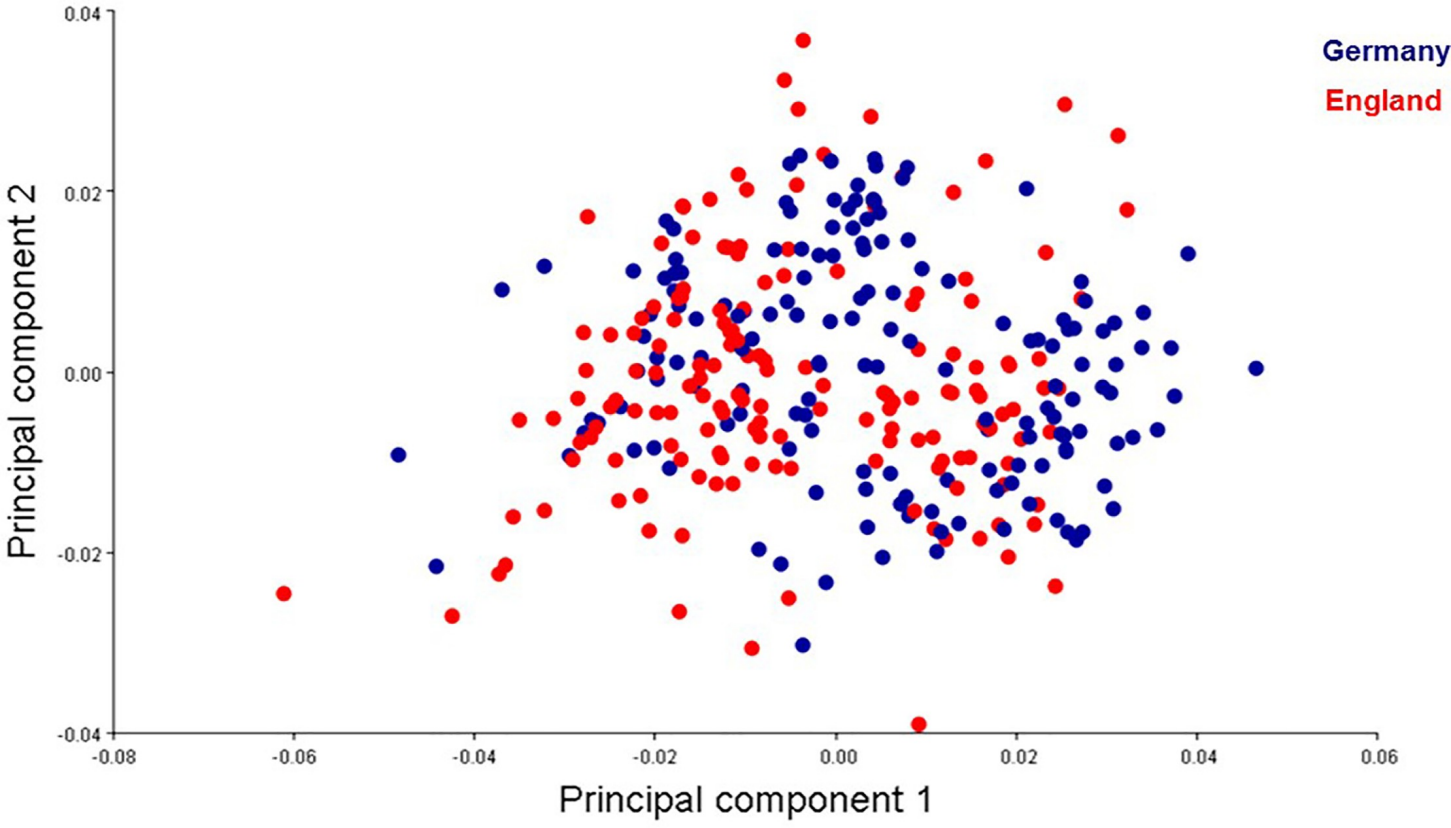
Projections of Procrustes-aligned landmark configurations on the first two principle components of the shape covariance matrix of *C*. *vicina* from Germany and England.

**Fig 6:**
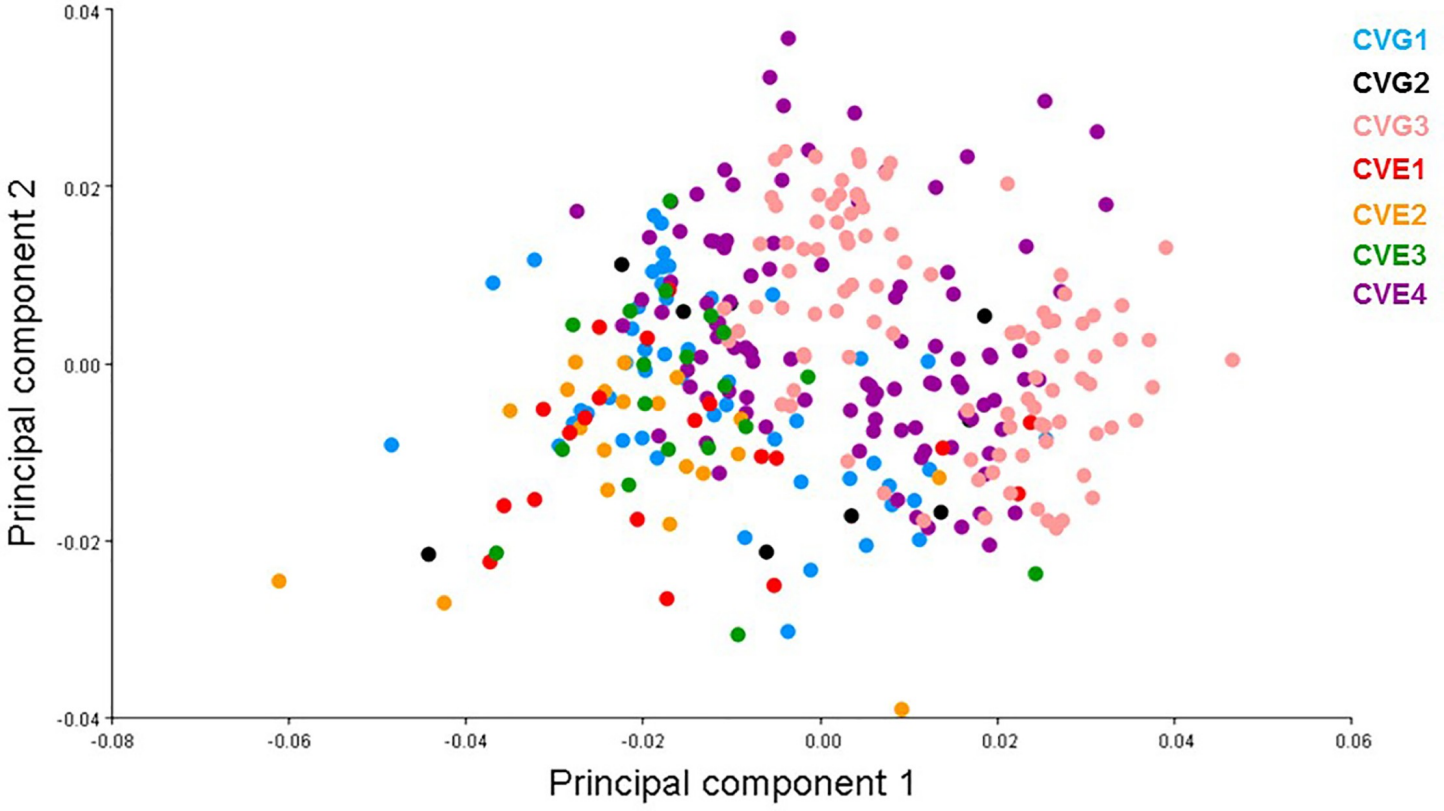
Projections of Procrustes-aligned landmark configurations on the first two principle components of the shape covariance matrix of *C*. *vicina* in each population, Germany (CVG1, CVG2, CVG3), England (CVE1, CVE2, CVE3, CVE4) see also Table 1.

**Fig 7:**
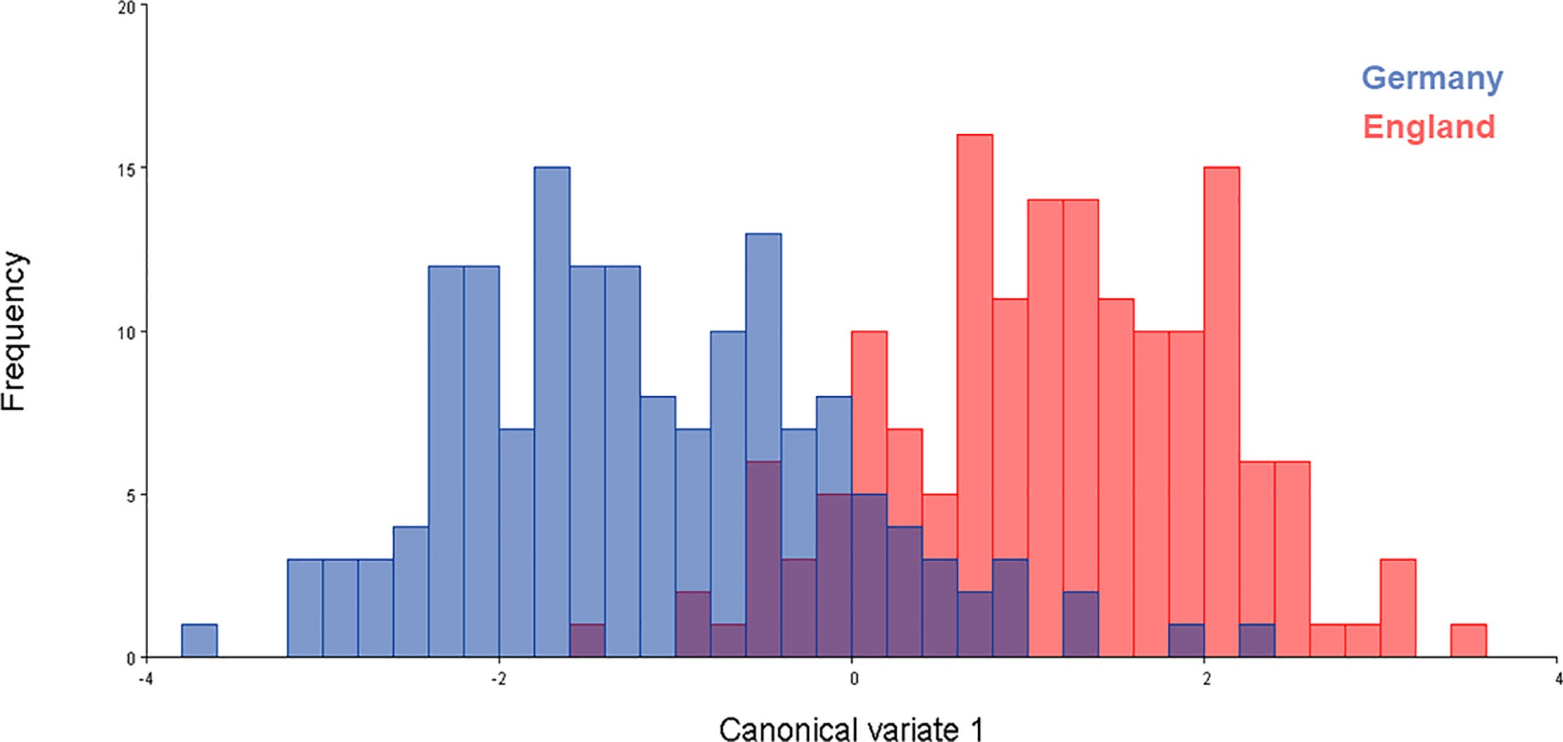
Frequency histogram of projected *C*. *vicina* wing landmark configurations from canonical variate analysis separating German and English population based on wing shape (variation among groups = 100%).

**Fig 8:**
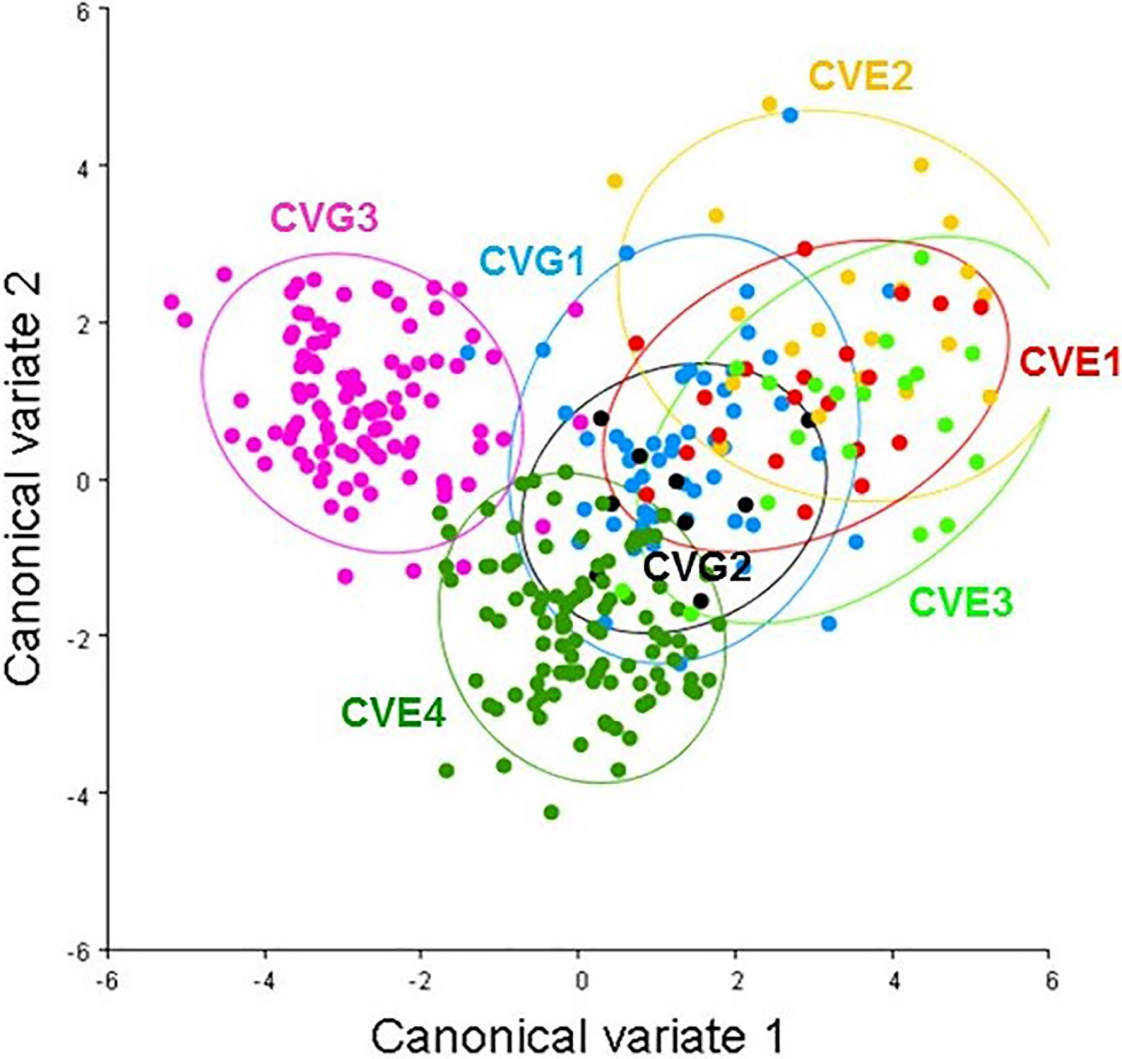
Scatter plot showing the distribution shape variable of *C*. *vicina* in each population along the first two canonical variant analysis (CV1 = 58.25%, CV2 = 23.24%) with 90% confidence ellipses; 7 locations representing as follow: Germany (CVG1, CVG2, CVG3), England (CVE1, CVE2, CVE3, CVE4).

PCA showed the shape variation between the two country populations (Germany and England; Fig 5; S2 Data) and between individual populations (CVG1, CVG2, CVG3, CVE1, CVE2, CVE3, CVE4; Fig 6; S2 Data) scattered on the first two principle component axes. For the two country populations, the total variation accounted for was 62.45%, i.e., the first component (PC1) accounted for 42.75% of total variation while the second (PC2) accounted for 19.70%. Projected configuration positions were close to one another in this space or even overlapped, indicating a similar average shape of the two populations (Fig 5). Nevertheless, the analysis from CVA showing the distribution shape variable of *C*. *vicina* in each country demonstrated two groups in the histogram, with 100% variation among groups (Fig 7; S2 Data). Comparison between all seven locations (CVG1-3, CVE1-4) demonstrated five groups of the distribution shape variable within a single generally overlapping area, excepting CVG3 and CVE4 (Fig 8; S2 Data). Mahalanobis distances and Procrustes distances of German and English *C*. *vicina* were 2.3065 (*P*<0.001) and 0.0122 (*P*<0.001). However, Procrustes distances between individual populations were also significantly different (*P*<0.001, *P*<0.05, *P*<0.01), except between CVG1-CVE3, CVG2-CVE1, CVE1-CVE2, CVE1-CVE3, and CVE2-CVE3 (Table 5; S2 Data).

**Table 5:** Difference in wing shapes of *C*. *vicina* from Germany and England analyzed with canonical variate analysis. Mahalanobis distances (above) and Procrustes distances (below).

Countries	Locations	CVG1	CVG2	CVG3	CVE1	CVE2	CVE3	CVE4
Germany	**CVG1**	-	4.5172***	4.4406***	3.3063***	3.6031***	3.7519***	3.1005***
	**CVG2**	0.0143*	-	5.7632***	5.5360***	5.6444***	4.6006***	4.6775***
	**CVG3**	0.0278***	0.0262***	-	5.9540***	6.4175***	6.5583***	4.0166***
England	**CVE1**	0.0120*	0.0173	0.0327***	-	2.6010*	3.5266**	4.4794***
	**CVE2**	0.0150**	0.0226**	0.0378***	0.0075	-	3.5194**	5.4276***
	**CVE3**	0.0096	0.0171*	0.0312***	0.0084	0.0105	-	4.8456***
	**CVE4**	0.0182***	0.0161**	0.0172***	0.0246***	0.0302***	0.0223***	-

*P*-values of significant differences denoted with asterisks (*** *P*<0.0001; ** *P*<0.01; *P<0.05).

The results of the DFA revealed significant difference of shape between the two countries and showed correct classification at 81% (cross-validation). DFA from wing shape between single groups was also significantly different (*P*<0.05), except for CVE1-CVE2, CVE1-CVE3 and CVE2-CVE3. Cross-validation tests showed 73–94% (CVG1), 67–89% (CVG2), 96–99% (CVG3), 55–90% (CVE1), 35–95% (CVE2), 47–89% (CVE3) and 95%-100% (CVE4), which indicated a high percentage of correct separation of CVG3 and CVE4 from other groups.

**Fig 9:**
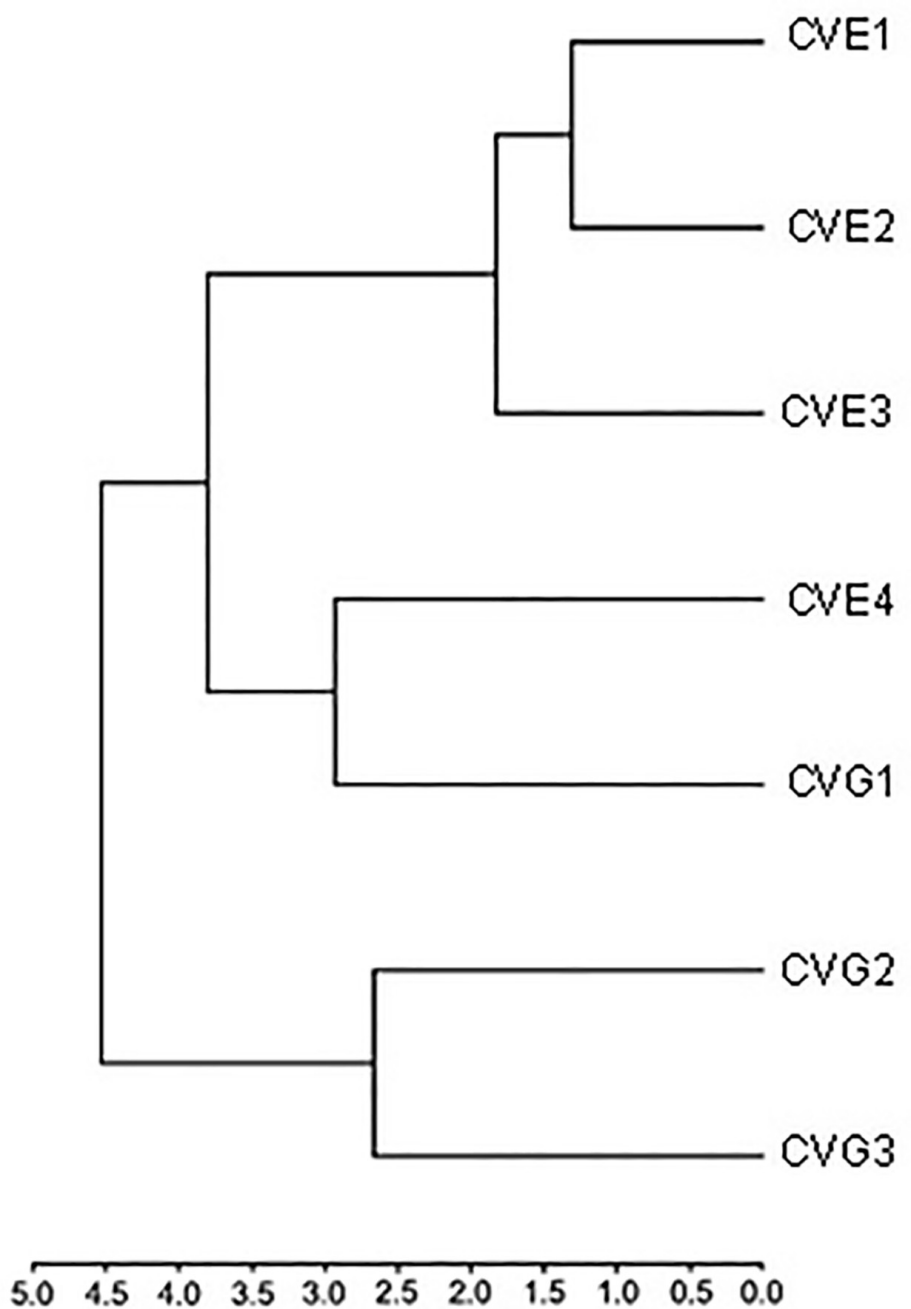
UPGMA dendrogram of *C*. *vicina* wing morphological proximity constructed based on the Mahalanobis distances from canonical variate analysis between Germany (CVG1, CVG2, CVG3) and England (CVE1, CVE2, CVE3, CVE4).

The UPGMA dendrogram of morphological similarity (Fig 9) placed CVG2 and CVG3 in one branch, while the remaining 5 locations were grouped in the other branch. This second branch showed two subdivisions, one with the populations from CVG1 and CVE4, and the other, more distant, with CVE1, CVE2 and CVE3, the latter in its own sub-branch (Fig 9; S2 Data).

### Phylogenetic analysis

**Fig 10:**
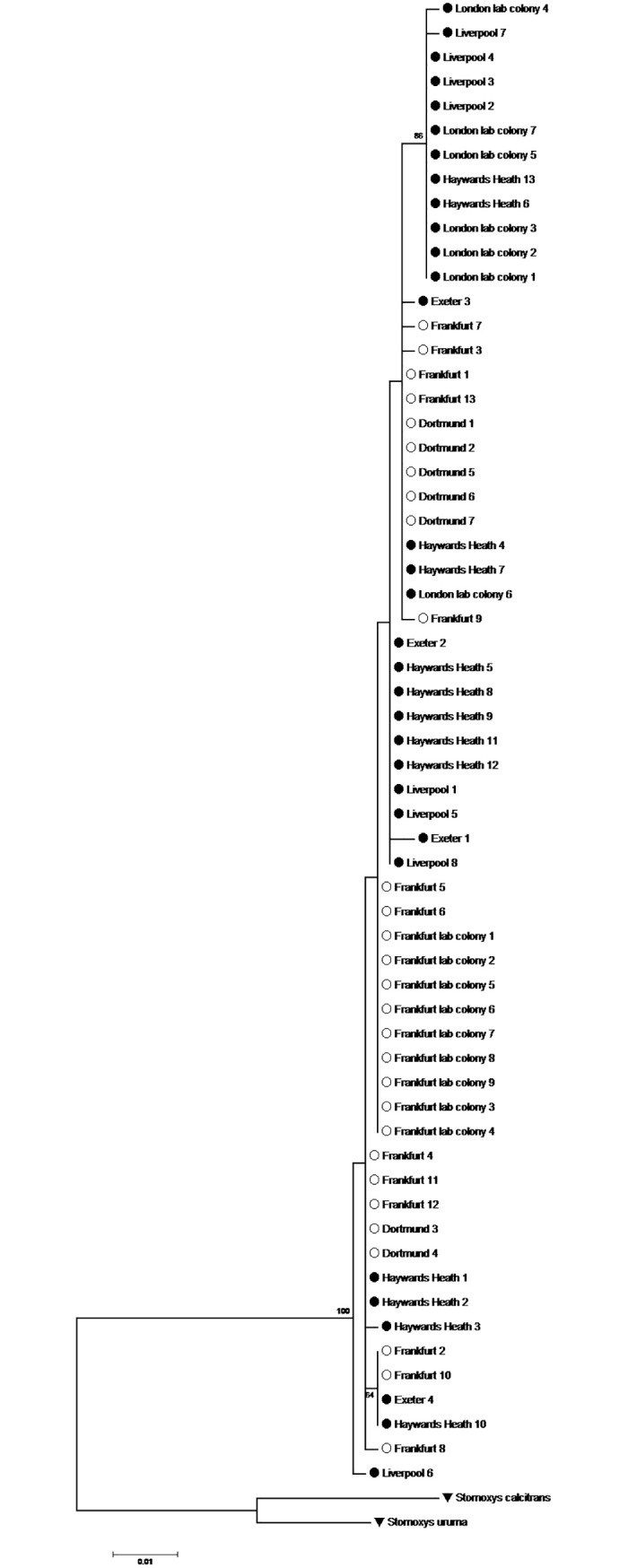
Molecular phylogenetic analysis by maximum likelihood method. The evolutionary history was inferred by using the maximum likelihood method based on the Tamura 3-parameter model [37]. There were a total of 528 positions in the final dataset. Evolutionary analyses were conducted in MEGA6 [36]. The symbol ○ indicates *C*. *vicina* samples from Germany, ● indicates *C*. *vicina* samples from England, and θ indicates outgroups.

784 bp’s of the cytochrome b gene were successfully sequenced and aligned for every analysed specimen. The nucleotide frequency of Adenine, Thymine, Cytosine, and Guanine was 36.65%, 36.65%, 13.35%, and 13.35%, respectively, which included 29 variable sites and 11 parsimony-informative sites. The ML tree based analyzed with the Tamura 3-parameter model showed an intermixture of German and English *C*. *vicina* in the same clade (Fig 10; S3 Data).

### Intraspecific divergence

The number of base substitutions was 0.4% on average for all German and English sites together. Additionally, distance analysis of intraspecific variation between each of the seven populations revealed low values (Table 6; S3 Data).

**Table 6:** Estimates of evolutionary divergence over sequence pairs between groups of *C*. *vicina* from the seven trapping locations.

Locations	Haywards Heath	Exeter	Liverpool	London Lab.	Frankfurt Lab.	Frankfurt
**Exeter**	0.4%					
**Liverpool**	0.4%	0.6%				
**London Lab.**	0.5%	0.7%	0.4%			
**Frankfurt Lab.**	0.3%	0.4%	0.6%	0.7%		
**Frankfurt**	0.4%	0.5%	0.5%	0.6%	0.3%	
**Dortmund**	0.2%	0.4%	0.4%	0.4%	0.3%	0.2%

Analyses were conducted using the Kimura 2-parameter (K2P) model [38]. The analysis involved 61 nucleotide sequences. Evolutionary analyses were conducted in MEGA6 [36].

## Discussion

In this study, geometric morphometric analysis of wing variation from CVA showed overlapping areas for almost every location. Procrustes distances revealed no variation of wing shape between Frankfurt am Main–Liverpool, Dortmund–Exeter, Exeter–Haywards Heath, Exeter–Liverpool, and Haywards Heath–Liverpool, whereas the DFA indicated the separation between two groups of observations between Exeter–Haywards Heath, Exeter–Liverpool, and Haywards Heath–Liverpool. The UPGMA dendrogram divided the populations into two groups, one from Germany and the other from England plus Frankfurt am Main (CVG1). In the latter group, the population from Frankfurt am Main was included within the same branch as the London laboratory colony. This might indicate that the German and English laboratory populations are converging towards a similar form and maybe mirror a potential effect of colonization in the laboratory—in a laboratory environment flight is not so important as in the field. Francoy et al. [39] compared the patterns of wing venation of the stingless bee, *Melipoua beecheii* (Hymenoptera: Meliponini), in Central America. The PCA results clearly indicated the formation of two separated clusters, one comprising the samples from Mexico and the other with the samples from Guatemala, El Salvador, Nicaragua, and Costa Rica. The dendrogram of morphological proximity also supports the existence of genetic lineages, with the population of Mexico in an isolated branch and the other populations sub-divided in another branch. However, using geometric morphometrics of wing shape of dwarf honey bees, *Apis florea* (Hymenoptera: Apidae), in four states of Iran demonstrated a high level of variation in this species between four states in Iran, with significant differences demonstrated by DFA [40]. Observation on PCA and CVA results showed populations of the blow fly *C*. *bezziana* were separated according to collection area, Africa (Tanzania, South Africa Sudan, Zaire, Zimbawe) and Asia (Sumba, Indonesia) [16] and another study using the same analysis found statistically significant differences in wing shape morphology between northern and southern populations of sand fly *Phlebotomus papatasi* (Diptera: Psychodidae) in Morocco [41]. Furthermore, wing morphometrics have been successfully used to discriminate between different insect species such as blow flies; *Cochliomyia* spp. [17] and *Amenia* spp. [42], sand flies [43], fruit fly; *Drosophila* spp. [44, 45], and parasitoid; *Eubazus* spp. [46]. Hence, wing morphometrics can be used as an additional tool for species identification and analysis of intra-specific variation of insects in the same species because it is relatively inexpensive, reliable, and easy to handle. However, non-damaged wings are required for analysis and it should be kept in mind that most morphometric studies focus on wing shape rather than wing size, because the latter can be easily affected by environmental factors [47, 48]. Wing shape has proven to be a more stable character than size and is more informative regarding the genetics and evolution of organisms [49, 50].

Wing morphometrics was compared here with a molecular phylogenetic approach by maximum likelihood method. *Calliphora vicina* populations all appeared in a single branch of the tree (Fig 10), with considerable mixing of locations in Germany (Frankfurt am Main, Dortmund, Frankfurt lab colony) and England (Exeter, Haywards Heath, Liverpool, London lab colony). This mixing was supported by the low percentage of intraspecific divergence observed between the seven sampling areas and between countries (Table 6). Hence, it was not possible to separate the populations from each other, with the exception of the Frankfurt and London laboratory colonies, which were relatively easy to distinguish from each other. The possible reason for this might be genetic drift and inbreeding, which occurred during establishment of the laboratory colonies, for many years in the German case, or have been a consequence of starting the colonies with more common alleles. Previous studies demonstrated that rare alleles are always lost after colonization and are replaced by increasing numbers of common alleles leading to a loss of variation [51–53].

We could not unequivocally discriminate *C*. *vicina* from different regions in Germany and England by using the cytochrome b gene. Similar results were observed in genetic variation studies on the blow fly *C*. *megacephala* in Malaysia (5 locations) using COI. Neighbour joining tree based on COI sequence showed two main groups, one branch comprised Penang and Selangor populations while another consisted of Johor, Pahang, and Sabah populations [54]. Including more genes in such analyses could be promising. May-Itza et al. [55] determined clear patterns of intraspecific variation within stingless bee species *Melipona beecheii* from Mexico, Guatemala, El Salvador, Nicaragua, and Costa Rica, using the internal transcribed spacer 1 of the ribosomal gene (ITS1). Furthermore, a strong differentiation between North American and West European populations of the blow fly *Phormia regina* was proven by analyzing the mitochondrial COI, COII, and cyt b genes [56] and clear patterns of intraspecific variation were also recorded with the Old World screwworm fly *C*. *bezziana* by analysis of nuclear and mitochondrial genes [57, 58]

Developmental study of both populations showed significant differences in larval length at certain time points, given as ADH, but is this difference of forensic relevance? Using an example of 1.2 cm larval length growing at 16°C reveals an ADH difference between German and English flies of about 130 (see Fig 3). Applying a lower development threshold of 0°C leads to a discrepancy of about 8 hours (130 ADH / 16°C = 8.1 h). Hence, an ADH discrepancy up to 192 at 16°C and 300 at 25°C will not exceed a 12 h window of time, and would enable estimates within an accuracy of one day, e.g., estimating the age of a German *C*. *vicina* population by use of the English data. The higher the temperatures are, the less the differences and discrepancies would be, as the impact of applying the lower developmental threshold becomes smaller. A mean time from egg hatch to the point of pupariation for both populations at 16°C, 25°C, and RT (average 21.5°C) was 11 days (3960 ADH), 7 days (4032 ADH), and 9 days (4428 ADH), respectively. According to Donovan et al. [4], where *C*. *vicina* growth rate was examined at temperatures between 4°C and 30°C, the minimum developmental temperature was estimated to be 1°C and 4700 ADH were required for development from egg to puparial stage. Furthermore, calculating the number of hours to develop to the puparial stage rearing at 5–29°C (average 13°C) from Wilson and Barnett’s formula showed 391.66 h (16.3 days) [59, 60] Similarly, other studies with *C*. *vicina* at 13°C [61] and 15.8°C [62] demonstrated 17.4 days and 12.25–18.33 days to develop from eggs to puparia. Using a quantile regression curve to establish developmental charts for *C*. *vicina* showed, for example, a 1.2 cm third instar larva which has grown at 25°C would be classified as 30–50 h old with a median age of 35 h [9]. In comparison, at the same larval length and rearing temperature the age estimation at 42.4 h (1060 ADH/ 25°C) in German flies and 41.6 h (1040 ADH/ 25°C) in English flies (see Fig 3), was within the 30–50 h range.

This study focused on whether or not populations of *C*. *vicina* show different rates of growth related to their geographical origin.Despite the fact that we recorded different developmental rates at some ADH landmarks, a calculation using the equation of Wilson and Barnett (59) gave for both populations a PMI_min_ estimation on the same day with a natural variation up to ±12 h. Nevertheless, this should not be the end of the story. Our findings highlight an important fact, the need to estimate the temperature threshold of a species, because using no or a wrong value could have dramatic consequences for the age estimation of a forensically important insect when applying the ADD/ADH method. Moreover, it also shows that we need more data on development at temperatures at the margins of development, as the impact of applying a lower threshold is greater at lower ambient temperatures. In times of climate change and extreme weather scenarios, not only lower thresholds of a species should be taken into account. *Calliphora* spp. could adapt to lower temperatures much better than warm-adapted taxa like *L*. *sericata* [3], but it might be impacted by varying temperatures. Larvae of Austrian *C*. *vicina* were observed to survive at high temperatures (35°C) but didn’t make it to pupation [5], whereas all larvae of a London *C*. *vicina*’s population died at this temperature [4]. Similar findings indicate that German *C*. *vicina* is not able to complete its development at a constant temperature of 29°C [60]. Analyzing a bigger range of temperatures and including more sampling times should be done to obtain more information to apply in forensic work. So far, the majority of developmental studies apply constant temperatures but reality, at least in the field, means fluctuating temperatures. Some forensically relevant flies (*C*. *vomitoria*, *Lucillia illustris*, *Sarcophaga argyrostoma*) have been demonstrated to develop more rapidly under fluctuating temperatures than at a constant temperature, if the temperatures do not exceed the optimal range for the organism [60].

Molecular phylogenetic analysis of *C*. *vicina* by the maximum likelihood method did not provide evidence for a consistent difference between the German and English country scale populations like the wing morphometric data did, although even the latter could not separate the different geographic populations from each other at a local level. Future studies using more genes, specimens and locations would be very worthwhile, producing molecular profiles which could serve as an indication of possible population differences useful in forensic case work.

## Supporting information

S1 Data Developmental study. (XLSX)

S2 Data Wing morphometrics. (TPS)

S3 Data Molecular analysis. (TXT)
